# Stressful Life Events and Inflammation in Midlife: Comparing Associations With suPAR, CRP, and IL-6

**DOI:** 10.1093/geroni/igaa057.805

**Published:** 2020-12-16

**Authors:** Kyle Bourassa, Line Rasmussen, Terrie Moffitt, Avshalom Caspi

**Affiliations:** 1 Duke University Medical Center, Durham, North Carolina, United States; 2 Duke University, Durham, North Carolina, United States

## Abstract

Stressful life events are associated with poorer health, but the physiological mechanisms of this association are unclear. One mechanism that might play a role in this association is systemic inflammation. Using participants (n=828) from the Dunedin Longitudinal Study, we studied the association of stressful life events and inflammation from age 32 to 45. Inflammation was assessed using C-reactive protein (CRP), interleukin-6 (IL-6), and suPAR. We examined associations between stressful life events and systemic inflammation, as well as whether adverse childhood experiences (ACEs) moderated these associations. More adult stressful life events were associated with greater suPAR at age 38 (r = 0.12, p < .001) and age 45 (r = 0.19, p < .001). CRP and IL-6 did not evidence consistent associations at age 38 and 45. An increase in suPAR from age 38 to 45 was associated with more stressful life events in the interim, β = 0.10, p = .001. SuPAR at age 45 was independently associated with both childhood ACEs, β = 0.20, p < .001, and adult stressful life events, β = 0.18, p < .001. ACEs significantly moderated the association of stressful life events and suPAR at age 45, β = 0.12, p = .001, such that people with more childhood ACEs evidenced a stronger association between stressful life events and inflammation in midlife. These results were robust to controlling for clinical (sex, body mass, smoking) and childhood covariates (childhood IQ, SES, self-control). Systemic inflammation is one mechanism through which stressful life events could impact health.

